# Diaphragm Design for an Electret Microphone Stethoscope

**DOI:** 10.1109/jsen.2025.3573907

**Published:** 2025-06-05

**Authors:** Alex Gaudio, Helena Hahn, James West, Mounya Elhilali

**Affiliations:** department of Electrical and Computer Engineering, Johns Hopkins University, Baltimore, MD 20218 USA

**Keywords:** Acoustics, auscultation, digital stethoscope, electret microphone, signal fidelity

## Abstract

This article investigates the design elements of a digital stethoscope and its signal fidelity in the presence of ambient noise. While the acoustic impedance matching literature demonstrates that signal pickup can improve when the diaphragm’s acoustic impedance closely matches that of the auscultated surface, this approach considers only one interface between two materials. Acoustic impedance matching with two materials may not explain the full picture for electret microphones, which introduce an air gap in their design and therefore have two interfaces between three materials. Introducing a hole in the electret stethoscope diaphragm reduces the three material impedance matching problem into a two material problem. Our empirical results with a 3-D printed electret stethoscope show that a hole in the diaphragm improves signal quality in a variety of tested settings. We additionally propose signal fidelity and noise leakage statistics, a signal quality score, and an amplitude spectrum, all based on the empirical distance correlation. We utilize these statistics to evaluate how all combinations of four diaphragm materials, six diaphragm thicknesses, and presence or absence of a hole affect signal quality of lung sounds in different ambient noise conditions on an acoustic phantom. Moreover, a result of this work is the fabrication of a high-quality and low-cost (U.S. $5) digital stethoscope that can be fabricated with a 3-D printer, soldering iron, electret microphone, and readily available materials.

## Graphical Abstract

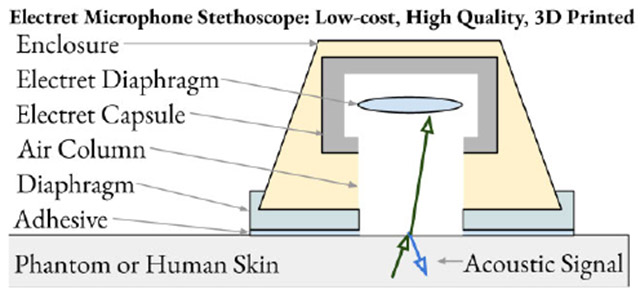

## I. Introduction

THE electronic stethoscope is an acoustic device for listening to body sounds. It incorporates a transducer that observes body sounds as electrical signals, an enclosure that attaches the device to the subject’s skin, and a system to electronically process the signals. The enclosure itself is comprised of three parts, each serving different functions: a material casing provides physical protection and ambient noise suppression; an internal acoustic chamber transmits the auscultated acoustic signal; and a diaphragm interfaces between the external sensed environment (e.g., human skin) and the internal chamber (e.g., air).

Transducers for digital stethoscopes include electret microphones [1], capacitive [2] and piezoelectric transducers [3], [4], microelectromechanical systems (MEMSs) [5], accelerometers [6], [7], [8], and other technologies reviewed in Section II. Electret microphones have the benefit of low cost, high sound quality, and reliable performance in different environmental conditions, but they are also sensitive to ambient noise.

Several studies have examined the influence of the enclosure’s three components on stethoscopes [9], [10], [11], [12], [13], [14], [15], [16], [17]. The literature, as surveyed in Section II, presents conflicting claims on whether a diaphragm attenuates or improves the signal. Some studies show that the absence of a diaphragm in a stethoscope with an air column improves signal amplitude [14] or reduces attenuation [15], [16], while others have shown the opposite in the presence of white noise [11]. Moreover, different diaphragm shapes also affect frequency response, and it has been shown that a nonuniform (convex shaped) diaphragm and a corrugated diaphragm have flatter frequency responses with higher amplitude than the diaphragms with a homogeneous and flat structure [14]. None of these works studied diaphragm performance in the presence of realistic ambient noise sounds. Furthermore, the metrics utilized for analysis are either simplistic or require qualitative interpretation of a plot. The design of acoustic impedance matched stethoscopes [2], [9], [18] introduces an objective to select a diaphragm material in order to transmit sound waves from the skin to the sensor with minimum signal loss. This literature utilizes the limiting assumption that there is only one interfacing surface between two materials, and those materials are the skin and the diaphragm. An electret stethoscope introduces two interfaces between three materials.

A main novelty of this work, and a hypothesis we evaluate, is that good signal quality can be obtained by exactly matching the acoustic impedance of the air column rather than approximately matching the acoustic impedance of the skin, and therefore reducing the number of interfacing surfaces from two to one. To the best of our knowledge, no existing works have studied a diaphragm with a through-hole design. A secondary novelty of our work is to study the effect of ambient noise, as captured from realistic hospital settings, on the diaphragm material and thickness. We have not identified any works that studied how the diaphragm affects the performance of an electret stethoscope (or of any stethoscope with two surface impedance matching problem) in the presence of realistic ambient noise, though Joyashiki and Wada [11] compared a polyurethane diaphragm to no diaphragm in the presence of white ambient noise. Our results agree with evidence from the literature that diaphragms may not be necessary in electret stethoscopes to detect signal with high fidelity, but we also show that a diaphragm with a through-hole in the center may mitigate ambient noise leakage due to the presence of sound attenuating material, while the through-hole design preserves signal fidelity.

This work studies the diaphragm and enclosure design for an electret microphone stethoscope that adheres to human skin via an adhesive. We conduct an ablative analysis comparing the signal fidelity to the noise leakage in order to identify an optimized diaphragm design. Independent variables include six diaphragm thicknesses (from 0.5 to 2 mm), four diaphragm materials, presence or not of a small hole in the diaphragm, six varying phantom signals, eight ambient signals, and three ambient noise gain levels. The tested materials include 3-D printed thermoplastic polyurethane (TPU), 3-D printed polylactic acid (PLA), 3-D printed biomedical resin (Elastic 50A) by Form Labs^1^, and cast resin polydimethylsiloxane (PDMS) by Sylgard^2^ 184 (Dow Inc.). All experiments are performed on an acoustic phantom in a sound booth. The outcomes of this study are.

1. *Material:* TPU offers the best noise suppression of the four tested materials and the best signal fidelity at higher ambient noise levels.
2. *Air column:* A diaphragm with a hole that allows direct contact between the internal air column and the skin or sensing medium gave better signal fidelity in most settings than a homogeneous diaphragm without a hole, though this finding is material dependent. Our results indicate that the diaphragm of an electret stethoscope both suppresses noise and attenuates the signal. TPU with a hole enabling direct contact of the air column with the skin or phantom is preferred over other tested diaphragm designs.
3. *Thickness:* There are no clear trends that summarize all evaluated materials. The influence of ambient noise leakage, possibly due to air gaps at interfaces between materials, has more significant impact on sensor quality than diaphragm thickness.
4. *Low cost and high quality:* A stethoscope can be constructed with 3-D printed materials, shielded audio wire, and an electret microphone capsule. The resulting device is low cost (U.S. $5), offers a high-quality signal, and can be assembled with a soldering station and 3-D printer.
5. *Signal fidelity and noise leakage statistics* based on the empirical distance correlations between a generated signal, generated ambient noise signal, and observed microphone signal are proposed to characterize the signal to noise ratio. We also propose a windowed distance correlation statistic to provide a statistical characterization of two signals across frequencies, and we introduce a quality measure to perform sensor selection.

Section II surveys stethoscope designs, impedance matching, air-coupled stethoscopes, and evaluation methodologies. Section III presents the experimental setup and evaluation methodology. Section IV shows and analyzes results. Section V discusses the scope, limitations, and future directions pertaining to this work.

## II. Background and Related Work

### Acoustic sensors used in stethoscopes:

The conventional or nonelectronic, stethoscope utilizes air-coupled sensors that can be as simple as a hollow wooden tube [19]. Conventional stethoscope designs transmit sounds observed on the skin surface, through the air and tubing of the device, and into the listener’s ear. Electronic stethoscopes [20] and wearable sound sensors [21] utilize transducers including electret, capacitive, optical, and piezoelectric microphones, as well as accelerometers used as microphones and triboelectric nanogenerators (TENGs).

Stethoscopes and their transducers should have a low noise floor, sensitivity to sounds in the low frequency range (approximately 50 Hz–3 kHz, assuming lung and heart sounds are of interest), and a general stability to environmental conditions like temperature and humidity. Preferably, the sensors should use environmentally friendly materials and have low manufacturing cost.

### Stethoscope sensors that are not air-coupled:

Stethoscopes made with piezoelectric microphone sensors [3], [4] and their miniaturized MEMSs variants, known as piezoelectric MEMS [5], utilize materials exhibiting the piezoelectric effect, such as ceramics and certain crystalline materials, to convert sound pressure into electricity. Piezoelectric sensors can have variability due to environmental temperature and typically lower sensitivity to low frequency sounds that has limited the popularity of these sensors for auscultation.

TENGs may enable wireless and battery-less stethoscopes [22], and recent work proposed a TENG with significantly higher sensitivity for auscultation than a piezoelectric sensor [23]. TENG approaches eliminate the air gap introduced by electret microphones, and they can be designed with environmentally friendly materials. TENG was invented in 2012 and research in this area is limited.

Accelerometers for recording sounds can be defined as a kind of piezoelectric sensor [6]. Accelerometers are well suited for the recording of low-frequency signals (such as 1 Hz–500 Hz), though they can measure higher frequencies [7]. The sensors are suitable for wearable devices and continuous monitoring [8] in part due to their insensitivity to ambient noise.

Impedance matched stethoscopes [9], [24] are designed to match the diaphragm’s acoustic impedance to that of human skin in order to minimize attenuation of the transmitted acoustic signal. The utility of impedance matching was recognized as early as 1980 in a piezoelectric stethoscope [10]. The capacitive stethoscope, as introduced with the patented ThinkLabs stethoscope [2], utilizes the stethoscope’s diaphragm as a dielectric polymer between two capacitive plates. The ThinkLabs diaphragm has a direct coupling to the skin, although it also has an air cavity behind the diaphragm. Impedance matching stethoscopes, by design, only address the signal transmission of one interface between two materials.

### Air-coupled and electret stethoscopes:

In contrast to previously described sensors, the electret microphone is air-coupled, which means acoustic vibration must travel through air to be observed by the transducer. The foil-electret microphone [1], [25] contains an air-coupled internal diaphragm (or charged dielectric film) that transforms acoustic vibrations in the air into measurable voltage differences, and the diaphragm vibrates freely inside an air cavity. In auscultation, transmitted body sounds must pass through human skin, through the stethoscope diaphragm, and finally into the air column inside of the capsule where it vibrates the electret microphone’s diaphragm. A signal passing from skin to sensor, therefore, has two opportunities to attenuate due to signal reflection caused by acoustic impedance mismatch, and therefore, the signal may undergo three or more reflections, or undergo other kinds of signal transmission challenges. An electret stethoscope, by design, introduces impedance mismatch at the interface between the skin and stethoscope diaphragm, from diaphragm to air column, and technically also from air column to the internal diaphragm.

### Electret stethoscopes and MEMS stethoscopes:

For continuous auscultation, Lee et al. [26] embedded a MEMS sensor into a soft flexible material for continuous wireless monitoring, with effort made to minimize the air gap. Joyashiki and Wada [11] compared an electret microphone with a urethane diaphragm that matches impedance of skin, an electret microphone without a diaphragm, and an accelerometer. The sensors were adhered to skin by covering them externally with adhesive tape, a solution that is prone to delamination of the seal between skin and device. They found that the urethane diaphragm was preferable to the absence of a diaphragm. Our work is similar, but we use a diaphragm with a small hole; the diaphragm serves to block noise yet permit transmission of the signal of interest.

### Diaphragm design for conventional stethoscopes:

Ertel et al. [12] studied the frequency response of 12 conventional nonelectronic stethoscopes, including the bell and tubing, and observed that all tested diaphragms attenuate the transmitted signal, but some more than others. The electret stethoscope is similar to a conventional stethoscope because both share a design challenge to match acoustic impedances between the skin, the stethoscope diaphragm and the air. A conventional stethoscope also introduces artifacts caused by the tubing. Wodicka et al. [13] analyzed the depth of the air cavity and found that larger cavity height diminishes the high-frequency response, therefore suggesting that air cavities with small height are preferable for lung sound analysis. Nowak and Nowak [14] studied seven diaphragms from commercially available conventional stethoscopes independently of an enclosure but affixed to the body. Their results show that the absence of a diaphragm gives higher amplitude in all frequency bands than all other tested diaphragms. None of these works rigorously studied the impact of realistic ambient noise on diaphragm design.

### Coupling of the chest and air-coupled stethoscope:

Nussbaumer and Agarwal [15] proposed a theoretical lumped-element model and experimental study of stethoscope acoustics that couples the stethoscope with the chest or phantom. They demonstrate that a diaphragm attenuates the signal at low frequencies, and that larger air cavity volume very slightly increases signal attenuation at low frequencies. An earlier work of Ertel et al. [16] also demonstrated that the stethoscope’s diaphragm attenuates the acoustic signal all 12 evaluated analog stethoscopes. Nowak and Nowak [17] studied the hypothesis that the stethoscope’s diaphragm can act as a low-pass or high-pass filter by varying the force pressing a stethoscope into a human subject’s skin. They concluded that an alteration to the amplitude spectrum is primarily due to the deformation of tissue under the stethoscope, and not from the deformation of the diaphragm itself. They also suggested that stiff diaphragms seem to have the best performance. Our work is complementary to these studies, where we emphasize the study of electret stethoscope design in the context of signal fidelity and noise leakage with a variety of realistic noise sounds from different hospital settings.

### Metrics for measuring stethoscope signal quality:

The reviewed literature utilizes a small variety of approaches to analyze signal quality. Curves visualizing the amplitude, magnitude, or power spectra across frequency bins are shown in nearly all papers reviewed, but an analysis of the relative quality of the signal is left to the reader’s qualitative and visual interpretation. Phase information available from the underlying Fourier transform is also largely ignored. In the presence of two signals, such as either the stethoscope recording and noise signals or the stethoscope recording and a ground truth phantom sound signals, the signal-to-noise ratio (SNR) offers a general framework for comparing the signals by reducing each to a scalar number, and computing a ratio. Peak SNR, used by Hui et al. [23], measures the SNR ratio between the peak amplitudes in two Fourier spectra, such as from the stethoscope and from the ambient noise. Peak SNR is a simplistic measure that can mislead researchers when the frequency response of the stethoscope is not flat, or when the noise signal does not represent all noise sources in the observed environment. However, the peak SNR can give an overall intuition for sensitivity.

The tissue-to-air ratio (TAR) [27] can be described as a kind of SNR that analyzes how an auscultated signal is distorted by an ambient noise signal, and the method offers a visual comparison of both signals’ transfer functions. The transfer functions are computed as the sound power level across frequency bins. Zanartu et al. [27] utilized TAR to show that air-coupled microphones are significantly improved when ambient noise is isolated from the auscultated signal. Joyashiki and Wada [11] utilized TAR to demonstrate that an electret microphone that is enclosed in 3-D-printed ABS and loosely attached to the skin by external tape leaks noise more than a comparable setup with a solid urethane diaphragm. Our evaluation is similar to [11] and [27] and actually demonstrates an opposite result because we utilize a solid diaphragm with a hole (and a 3-D printed enclosure) that properly seals out ambient noise while permitting transmission of the auscultated signal. The magnitude squared coherence (MSC), as adopted by Rennoll et al. [9], gives an estimate for how well one signal can be linearly transformed into another, and it computes a ratio comparing the cross spectral density of the signals to the product of the respective power spectral densities. Since cross-spectral density is estimated by sampling frequency bins, the MSC depends on the bandwidth (and range) of the sampled bins, and suffers from need to choose the bin width. The MSC approaches zero when the cross-spectral density approaches zero, and this does not measure general statistical independence. Pearson correlation is an alternative measure to compare two signals. Semmlow [28] utilized it in the time domain to compare the auscultated signal to the transmitted signal. The Pearson correlation of two time-domain signals only measures a linear correlation between two signals, and it can give a large response to nonlinear distortions that have minor effect on signal fidelity. The empirical distance correlation [29], generalizes the Pearson correlation to represent linear and nonlinear relationships, and it also analyzes either paired random vectors or multiple observations of paired random vectors. Comparisons with Pearson correlation have shown that the distance correlation is a superior metric [30], [31], but the statistic is relatively new (2007), and not yet well utilized for signal analysis. This work employs the empirical distance correlation to define signal fidelity and noise leakage statistics.

## III. Methods

**Fig. 1.**
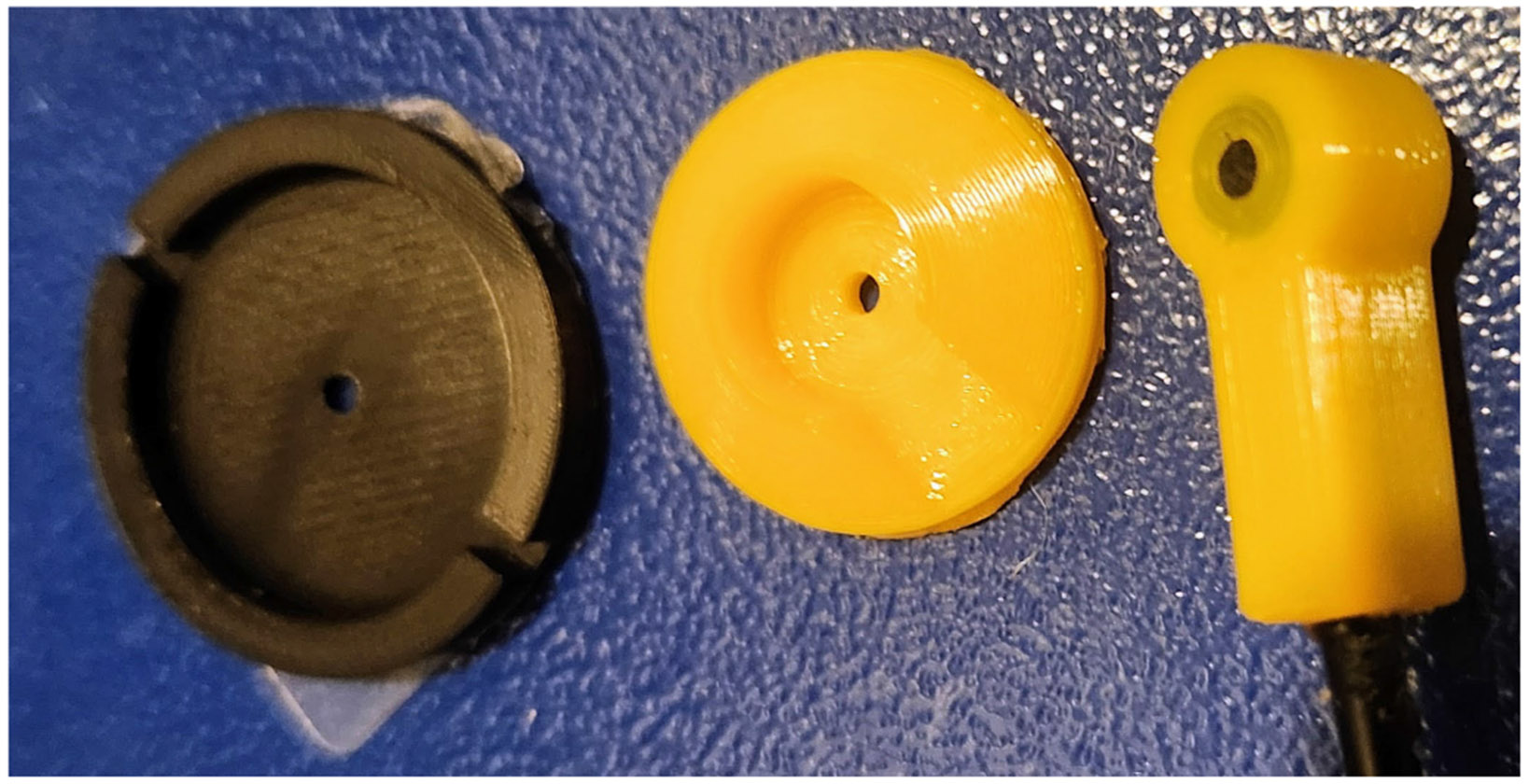
Stethoscope sensor designed with 3-D printed components. The enclosure is 3-D printed in TPU (yellow, in two parts). The example diaphragm (PLA, in black) attaches to the middle component to form an interchangeable diaphragm. The small tab attached to the black diaphragm is a protective film covering the double-sided tape.

**Fig. 2.**
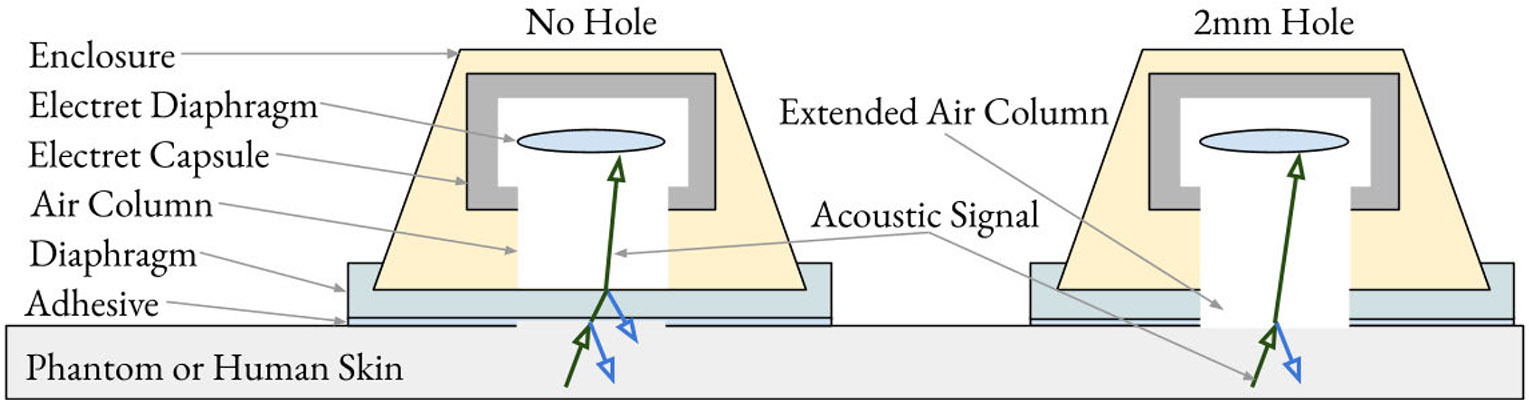
Digital stethoscope construction using an electret microphone imposes an air gap between the electret’s diaphragm and either the stethoscope diaphragm or the sensing medium. In the 2-mm hole design (right), the air column interfaces directly with skin, and the figure shows less opportunity for reflection of the acoustic signal.

**Fig. 3.**
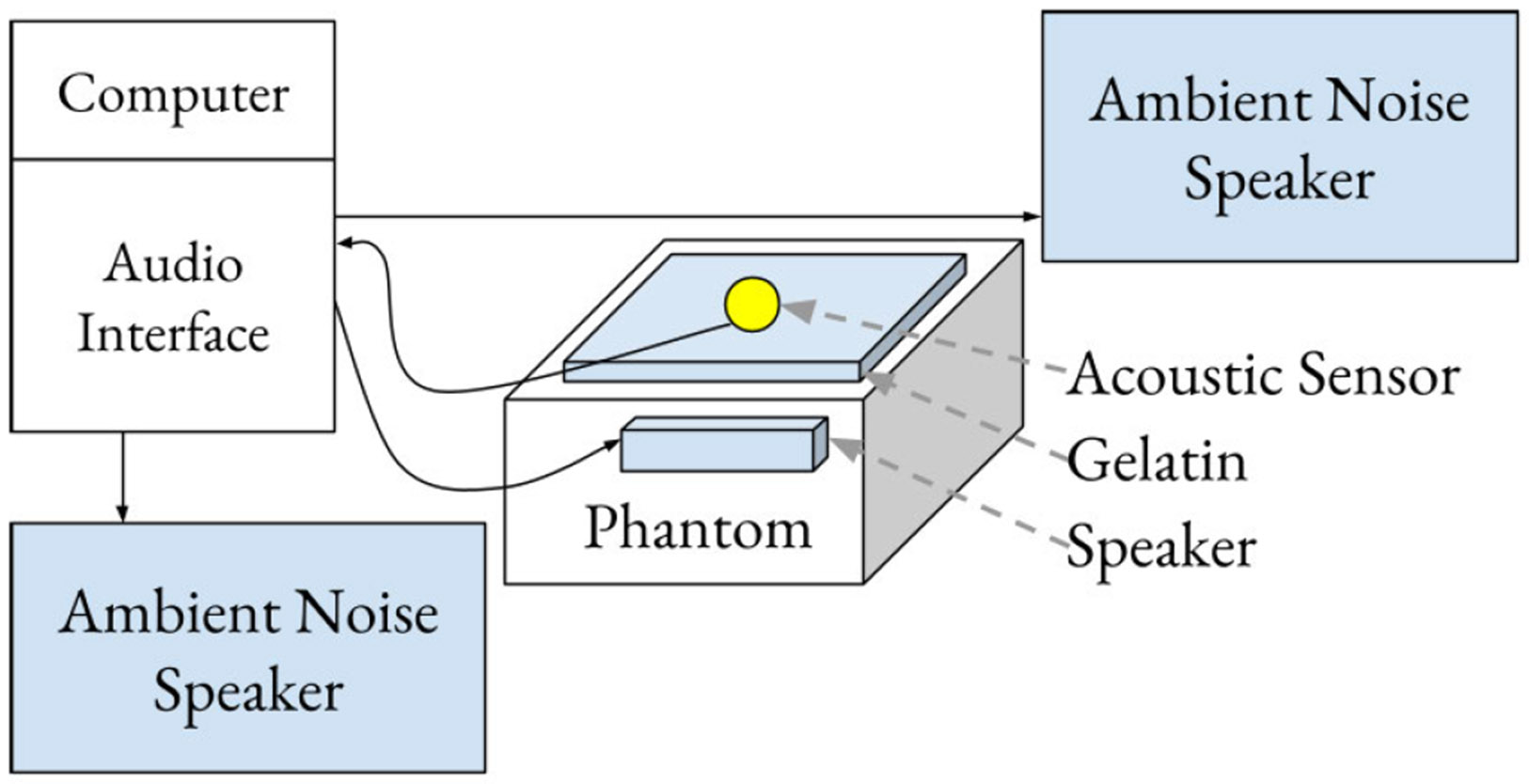
Illustration of the experiment setup in a sound booth. The phantom and ambient noise speakers playback audio signals p and s sent from the computer, and the sensor transmits observed recorded signal r back to the computer.

A set of experiments was defined to evaluate how diaphragm material, thickness, and through-hole design affect the recording of body sounds from an acoustic phantom in the presence of ambient noise. Figs. 1 and 2 show the design of the acoustic sensor and Fig. 3 shows the setup of a sound booth in which experiments were conducted.

### A. Experiment Setup, Sound Booth

All components shown in Fig. 3 were located inside an acoustically isolated sound booth except the computer and human operator. We utilized the same phantom described in [18], with a fresh gelatin layer (Humimic Medical, Gelatin 2) of thickness 11 cm and created following the procedure in the supplementary of [18]. For a given sensor, there are multiple experiments, and each experiment contains three simultaneous and synchronized acoustic events: an ambient noise signal is emitted on ambient noise speakers at a given gain, a phantom signal is emitted on a phantom speaker at a constant gain, and a recorded signal is received from the stethoscope sensor through a dedicated audio interface. The sample rate of all signals is 44 100 Hz. The ambient speakers are equidistant and approximately 65 cm from the phantom. The phantom signals are represented as a set 𝒫 containing six lung sound vectors, where each signal vector p∈𝒫 is 5 s long. The lung sounds were manually selected from digital stethoscope recordings in the ICBHI respiratory sound database [32] and the PERCH database [33]. Nine noise sounds are represented as a set 𝒮, where each signal s∈𝒮 is also a five seconds long vector. Six of the nine noise sounds were curated from the PERCH study and from a private collection of ambient noise recordings. The private recordings were collected in a clinical environment where auscultation was performed, both at the Johns Hopkins Hospital in Baltimore, Maryland (US) and in the Kamazu Central Hospital in Lilongwe (Malawi). The three manually generated noise sounds were uniform noise, pink noise, and silence. A set 𝒱 of three ambient noise gain levels is denoted 𝒱≜{(1∕40),(1∕10),(1∕2)}. The Cartesian product of all three sets identifies 135 pairs of emitted signals necessary to perform 135 experiments of a given sensor. Both signal vectors s and p in each experiment are each normalized by a function $n_{y}(x)=(yx∕\max\;x)$, which converts values of each signal vector into the range of [0,1] and multiplies by a given gain level y. The pair of normalized output signals for each ith experiment are therefore denoted as {(n(1∕40)(p), $n_{v}(s))$; ${\left(p,s,v_{i}\right)}_{i}\in 𝒫\times 𝒮\times 𝒱\}$. The normalized noise signals have an expected sound pressure levels of 57, 68, and 79 A-weighted decibels (dBA) for respective gains (1/40), (1/10), and (1/2), where the expectation had a standard deviation of ±10 dbA in each of the three cases. The sound pressure level measurement was performed with a Martel 322 Sound Level Meter tool positioned at the location on the phantom where the stethoscope sensor would be. For each normalized pair, a corresponding recording from the evaluated stethoscope sensor is obtained and denoted as $r_{i}\in ℛ$. The set ℛ contains 135 corresponding recordings. The inputs and outputs of all experiments for a given sensor are denoted

(1) ℰ≜{(n140(p),nv(s),r);}{(p,s,v)i∈(𝒫×𝒮×𝒱),ri∈ℛ}.

### B. Experiment Setup, Sensor

The 135 experiments in the set ℰ were performed once for each sensor, and 32 sensors were evaluated. Sensors vary in material type (m), thickness (t), and whether they have a hole in the center of 2 mm in diameter (h). These properties are identified with subscripts ${ℰ}_{m,t,h}$. We therefore define a superset of all experiments across all sensors

(2) $$\{{ℰ}_{m,t,h};\;\;m\in \{\text{TPU},\text{PLA},\text{Elastic50a},\text{PDMS}\}\;,\;\;t\in \{0.5,1.0,1.5,2.0\}\;,h\in \{0,1\}\}\;.$$

Diaphragm materials are identified as m∈{TPU,PLA,Elastic50a,and PDMS}. TPU is a TPU 3-D printer filament (overture^1^ TPU, in yellow). PLA is polylactic acid filament (PolyTerra^2^ PLA from Polymaker^1^, in black). The TPU and PLA materials were printed on Bambu Lab^1^ X1C 3-D printer with a 0.4 mm nozzle, 0.2 mm layer height, and 100% concentric infill. The nozzle temperature was 250 °C for TPU and 220 °C for PLA. Elastic50a is a proprietary biomedical grade resin (Biomed Elastic50A) from Form Labs that was printed directly on the Form Labs build plate, without support. PDMS is a PDMS cast resin (Sylgard 184, Dow Inc.^1^) prepared with a 1:10 ratio of parts A and B. While the other materials consist of a cylindrical disk with raised side walls and a small overhang, the PDMS, which was cast rather than 3-D printed, was simply a cylindrical disk, and a retaining ring with the raised side walls with overhang was 3-D printed in TPU. The PDMS diaphragm was contained inside a TPU retaining ring due to the inability to cast the PDMS into the desired shape. Due to the retaining ring, the diameter of the PDMS diaphragm was smaller by 2 mm. As shown in Fig. 1, the diaphragm (left side) attaches to the middle part of the enclosure, and the middle part attaches to the right-most part.

The diaphragm thicknesses, in millimeters, are d∈{0.5,1.0,1.5,2.0}. In practice, the PDMS diaphragm thicknesses varied to within about 0.3 mm, and more accurate measurement of the thickness is challenging due to the flexibility of the material.

The presence of the 2 mm hole is indicated as h∈{‘‘No Hole’’,‘‘2mm Hole’’}. Fig. 2 shows that the 2 mm hole extends from the opening of the capsule directly to the phantom or skin.

#### Adhesive:

A double-sided adhesive (3M^2^ 2477P) adheres the diaphragm to the phantom or skin. The adhesive is 0.4 mm thick. Because the phantom’s gelatin layer did not stick securely to the adhesive, it was necessary to apply a 50 g weight on top of the sensor that ensured the seal between sensor and phantom was not broken by any twist in the audio wire leading into the sensor. The adhesive was laser cut into the shape of a donut so that it always had a hole. The adhesive hole diameter was 6 mm, which is larger than the 2 mm air column. The purpose of the larger diameter was to ensure the skin or phantom can make contact with the “No Hole” diaphragms in at least a 2 mm region.

#### Enclosure and dimensions:

All sensors shared the same 3-D printed enclosure, shown in Figs. 1 and 2. The enclosure was 3-D printed in TPU with the same 3-D printer configuration described for the diaphragms. The electret capsule (Soberton EM6027) is press-fit inside the enclosure, its rear vent hole and wire leads encapsulated in silicone glue, and a TPU cap is pressed into the wet glue. The height of the cylindrical air column from the capsule’s aluminum casing to the diaphragm was approximately 2.5 mm. The diaphragm diameter was 28 mm. The PDMS inside its TPU retaining ring was 15 mm in diameter.

Fig. 2 shows how the acoustic signal may reflect or transmit at transition points between skin, diaphragm, and air. Each transition point may be subjected to undesirable acoustic transformations. For instance, external noise may enter through the diaphragm, or the auscultated signal may be reflected away from the sensor. Moreover, interactions caused by the transition points between these materials may result in multiple reflections, further modifying the signal. The diaphragm with a hole, shown on the right side of the figure, has less opportunity for sound to reflect away from the sensor.

### C. Evaluation

#### Signals as random variables:

Any single experiment in the set defined in (1) contains a tuple of three signal vectors: the normalized phantom signal n(1∕40)(p), the normalized noise signal $n_{v}(s)$, and the recorded signal r. The observation of each signal vector can be interpreted as a probability event. Since there are multiple observations of each probability event with any given set experiments ${ℰ}_{m,t,h}$ for the sensor (m, t, h), each of the three respective signal vectors can be represented as a statistical random variable with its own probability distribution.

#### Distance correlation:

We desire three properties in any given experiment: 1) statistical independence between the recorded signal r and ambient noise signal $n_{v}(s)$; 2) statistical dependence between r and the phantom signal n(1∕40)(p); and 3) statistical independence between $n_{v}(s)$ and n(1∕40)(p). Statistical independence between r and $n_{v}(s)$ means that no knowledge of the value of r can be gained by knowledge of the value of $n_{v}(s)$. Statistical dependence is not as well defined, and we will adopt linear dependence as a proxy, defined as the equality of one signal to the other after an affine transform. Linear dependence is limited by an assumption of a particular representation space, such as the time domain or frequency domain. We desire all three properties 1)–3) to hold for all experiments in any given set ${ℰ}_{m,t,h}$.

The empirical distance correlation [29] between paired samples of two statistical random variables, or signals, quantifies the degree of statistical independence versus equality through affine transform, and is useful to analyze sensor performance. The statistic ranges from [0, 1], where a value of 0 means the two signals are statistically independent, and a value of 1 means that one signal transforms into the other by means of an affine transform. In this work, the correlated signals are subject to imperfect measurement conditions. Therefore, we assume that a value of 0 implies approximate independence rather than true independence. Since the time and frequency domains are both relevant in our analysis, we compute distance correlations of time-domain signals, and also of the Fourier magnitude spectra of the signals. For the magnitude spectra, only the frequency bands between 0 Hz and 4 kHz are considered. The following simplified notation is utilized: ${\mathrm{dcor}}_{m,t,h,\text{time}}(s_{v},r_{v})$ evaluates the statistic for each of the normalized ambient noise vectors $\left\{n_{v}(s);s\;\in \;𝒮,v\;\in \;𝒱\right\}$ and the corresponding recordings $r_{v}\;\in \;ℛ$; the ${\mathrm{dcor}}_{m,t,h,\text{time}}(p,r_{v})$ compares the normalized phantom signals to the recorded signals. The subscript v ensures the distance correlation is computed for each ambient noise gain. Replacing time-domain signals with Fourier amplitude spectra, we similarly define ${\mathrm{dcor}}_{m,t,h,\text{freq}}(x,r_{v})$ where x is either p or $s_{v}$ as before.

#### Quality score for sensor selection:

A stethoscope sensor should maximize ${\mathrm{dcor}}_{m,t,h,\text{freq}}(p,r_{v})\in [0,1]$ and minimize ${\mathrm{dcor}}_{m,t,h,\text{freq}}(s_{v},r_{v})\;\in \;[0,1]$ for all ambient noise gains v∈𝒱. We can transform the objective into a maximization by considering a 2-D rectangular region between the origin and point (1, 1) formed by ${\mathrm{dcor}}_{m,t,h,\text{freq}}(p,r_{v})$ and $1-{\mathrm{dcor}}_{m,t,h,\text{freq}}(s_{v},r_{v})$. Since there are multiple gains v∈𝒱, we average each term overall v in (3). The optimal sensor, represented as a point p in this region, should be maximally close to the point (1, 1). Taking a geometric approach, we want to maximize the area under the rectangle formed by p and the origin. We therefore aim to choose the independent variables (m, t, s) that maximize

(3) $$Q_{m,t,h,\text{freq}}≜xy,\;\text{where}\;\;x≜\frac{1}{|𝒱|}\sum_{v\in 𝒱}(1-{\mathrm{dcor}}_{m,t,h,\text{freq}}\left(s_{v},r_{v})\right)\;\;y≜\frac{1}{|𝒱|}\sum_{v\in 𝒱}{\mathrm{dcor}}_{m,t,h,\text{freq}}\;(p,r_{v})\;.$$

**Fig. 4.**
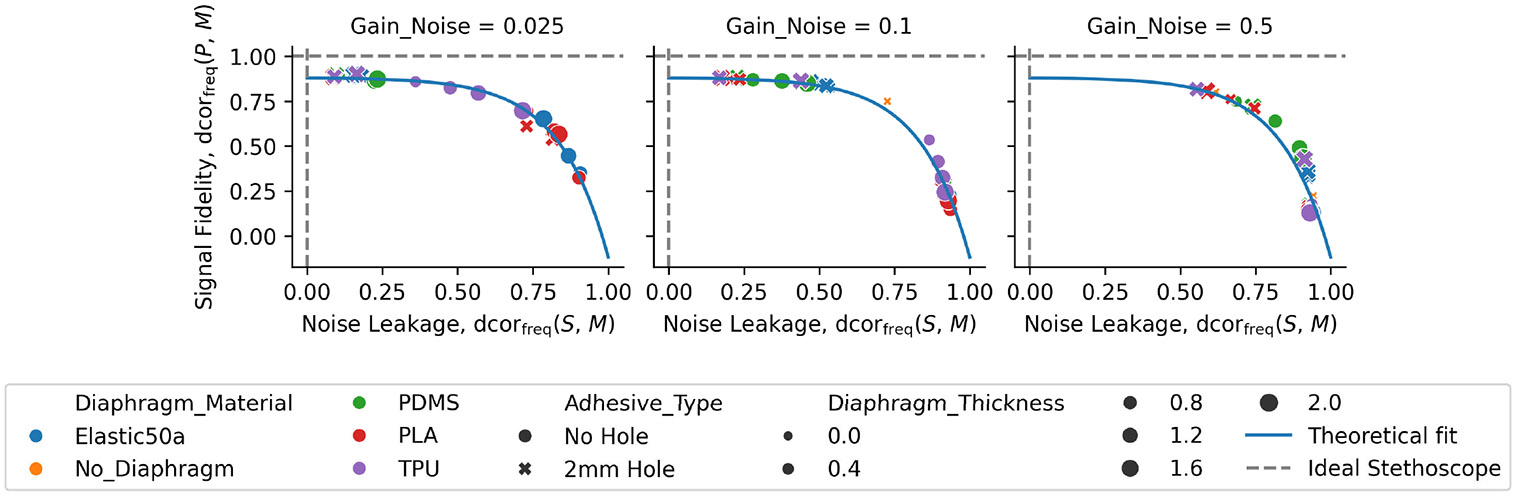
Signal fidelity versus noise leakage at varying levels of ambient noise provides an analysis space based on distance correlation. In each plot, the optimal sensor would appear in the top left of each plot and is linearly correlated with the amplitude spectrum of the phantom signal and statistically independent of the amplitude spectrum of the ambient noise signal. Each point represents an evaluation of 135 experiments with a single sensor. See Table I for the aggregated results of individual sensors in table form. A theoretical fit line shows that all sensors can be described by an exponential function in the space.

While the sensor quality score (3) is similar in spirit to a SNR due to the multiplication of two terms, it has better numerical stability because it is bounded inside [0, 1] while the SNR can have exploding values or division by zero. Moreover, the quality metric defines a Pareto boundary, where two sensors may have equal quality, or equal area, but one may have higher signal fidelity, while the other has lower noise leakage. The Pareto boundary follows a diagonal line from top left to bottom right of the rectangular region. Note that the rectangular region can be considered in Fig. 4 if the x-axis is flipped (1−x).

#### Windowed distance correlation and windowed quality score:

We propose windowed distance correlation over an amplitude spectrum of two signals. A set of frequency bands can be defined on an amplitude spectrum representation. Each frequency band f can be represented as either a single amplitude or a vector of amplitudes selected from a subset of the fast Fourier transform. For each frequency band f, a distance correlation is computed on the subset of a Fourier amplitude spectrum contained inside the given band, denoted ${\mathrm{dcor}}_{m,t,h,freq}({\mathrm{fft}}_{f}(\cdot ),{\mathrm{fft}}_{f}(r_{v}))$, where · is replaced by the random variables representing the ambient noise $s_{v}$ or phantom p signals.

It is reasonable to choose a sensor using a quality metric based on human hearing. The Bark frequency scale [34] identifies the critical bands of human hearing. In the range of less than 4 kHz, the start frequencies of the bands can be defined as [20, 100, 200, 300, 400, 510, 630, 770, 920, 1080, 1270, 1480, 1720, 2000, 2320, 2700, 3150, 3700,] Hz, and the corresponding bandwidths are [80, 100, 100, 100, 110, 120, 140, 150, 160, 190, 210, 240, 280, 320, 380, 450, 550, 700] Hz. For each of these bands, an amplitude spectrum is extracted from the fast Fourier transform, allowing the computation of the windowed distance correlation at band f.

Using the notation f to identify each Bark critical band and its corresponding bandwidth, we define one sensor quality score for each frequency band f

(4) Qm,t,h,bark,f≜xy,wherex≜1∣𝒱∣∑v∈𝒱(1−dcorm,t,h,freq(fftf(sv),fftf(rv))y≜1∣𝒱∣∑v∈𝒱dcorm,t,h,freq(fftf(p),fftf(rv)).

To aggregate the windowed Bark score across frequency bands, we also define $Q_{m,t,h,\text{bark}}=(1∕F)\sum_{f}Q_{m,t,h,\text{bark},f}$ as the average quality over the F=18 defined Bark critical bands. Note that $Q_{m,t,h,\text{freq}}$ in (3) is not windowed and does not use an average in this way. In principle, it would be desirable if the values of $Q_{m,t,h,\text{bark}}$ and of $Q_{m,t,h,\text{freq}}$ agreed in rank over all sensors (m, t, h).

*SNR:* We also utilize a widely utilized SNR metric in order to validate general agreement to the distance correlation, ${\mathrm{dcor}}_{m,t,h,\text{freq}}$. The SNR can be defined as ratio of two scalar terms. For each experiment parameterized by (m, t, h), we compute

(5) $${\text{SNR}}_{m,t,h}=10\;\log_{10}\left(\frac{{\left\|\mathrm{fft}\;(r_{v})\;\right\|}^{2}}{{\left\|\mathrm{fft}\;(n_{v}\;(s))\;\right\|}^{2}}\right).$$

### D. Measurement of Acoustic Impedance

A separate set of experiments were performed to analyze the influence of acoustic impedance matching on diaphragm material selection. For each tested material (Elastic50a, PDMS, PLA, TPU), a solid disk (42 mm diameter, 10 mm height) was fabricated with identical 3-D printer settings used for the diaphragms. The PDMS sample height was 5 mm. The impedance was measured using the through-transmission technique with the same equipment and nearly the same setup described in supplementary material of Rennoll et al. [9]. A 10-gallon fish tank wrapped in plastic-wrap to isolate ultrasonic noise was filled with water. Ultrasonic receiver and transmitter probes were submerged on opposite sides of the tank and supported by a welded metal frame. A thermometer measured the water temperature, which is used to calculate the speed of sound in water [35] via the equation $v_{\text{water}}(t)=1404.3+4.7\;t-0.04\;t^{2}$, where t is temperature in degrees Celsius. A plexiglass barrier, with a hole to transmit the ultrasonic signal and spring mechanism to optionally hold a test sample in front of the hole, was positioned close to the receiver probe. A function generator emitted a 0.5-MHz sine wave burst signal (with 30 cycles and 10 volt peak-to-peak amplitude). The generated signal transmitted directly to an oscilloscope, as well as to the ultrasound probe. The received ultrasound signal was observed in the oscilloscope’s second channel. A time offset between the generated signal and the onset of the observed peak signal was recorded multiple times, including when only water existed between the probes (baseline), and also with water and different tested materials between the probes. The difference of the observed offsets between the only water baseline and other materials gives a time shift δt, and the acoustic velocity of the evaluated material can be determined via the following equation:

(6) $$v=\frac{h}{\delta t+\frac{h}{v_{\text{water}}}}$$

(7) z=ρv.

The material volumetric mass density was also computed via the suspension technique [9], [36]. Last, specific characteristic acoustic impedance was computed via (7), where h is the height of the tested material.

## IV. Results

The choice of diaphragm significantly affects the signal quality, as shown in Fig. 4. The figure compares signal fidelity (y-axis) to noise leakage (x-axis), and shows a clear relationship between the two axes. The ideal stethoscope has a signal fidelity of 1.0 and noise leakage of 0.0 (top left corner of plot, identified with dashed gray lines), and the worst-possible stethoscope has the opposite (bottom right corner of plot). Each evaluated sensor appears as a point (x, y) in the space. The points fall along an exponential curve that we heuristically modeled with the function $f(x)=1-e^{w_{0}(x-1)}+w_{1}$ and solved using a least-squares objective

(8) $$\text{arg}\;\underset{w}{\text{min}}\sum_{\left(x,y\right)}{|\left(1-\left(e^{w_{0}(x-1)}+w_{1}\right)\right)-y|}^{2}$$

where the parameters obtained were w=[6.196,0.119] after initialization at w=[1,2]. The bias value of $w_{1}=0.119$ shows that the maximum signal fidelity modeled by this curve would be at 0.88. It is not clear if the bias is a result of external noise in the sound booth or other experimental conditions that cause signal distortion. The exponential relationship between signal fidelity and noise leakage also demonstrates that a sensor with a good signal is also good at blocking noise. The fact that the data are well modeled by an exponential curve suggests there was minimal amount of uncontrolled noise in the study. Moreover, an underlying assumption of the experiments is that the phantom signal is statistically independent of the ambient noise signal, and in support of this assumption, we observe that ${\mathrm{dcor}}_{\text{freq}}(p,s_{v})$ equals zero in all experiments to at least eight decimals. From the figure, it is clear that the material, the presence of a hole, and the ambient noise level have significant influence on performance, while diaphragm thickness is less important.

Table I summarizes all points in Fig. 4 for the purpose of material selection. Note that the table demonstrates the agreement in rank of the quality scores derived from the frequency representation and the time-domain representation. The quality scores are described in (3). The results in the table were averaged across diaphragm thickness and ambient gain levels.

TPU with a 2 mm hole is the diaphragm with the highest quality overall, as shown in Table I. Moreover, removing the diaphragm and using the TPU enclosure alone with an adhesive layer directly applied to the enclosure results in better performance. With no diaphragm, the height of the air column between sensor and skin is smaller, and there is no opportunity for noise to enter at the seam between the diaphragm and enclosure. Because the noise leakage statistic is close to one for the diaphragms with no hole, the results in this table verify the importance of noise isolation.

**Fig. 5:**
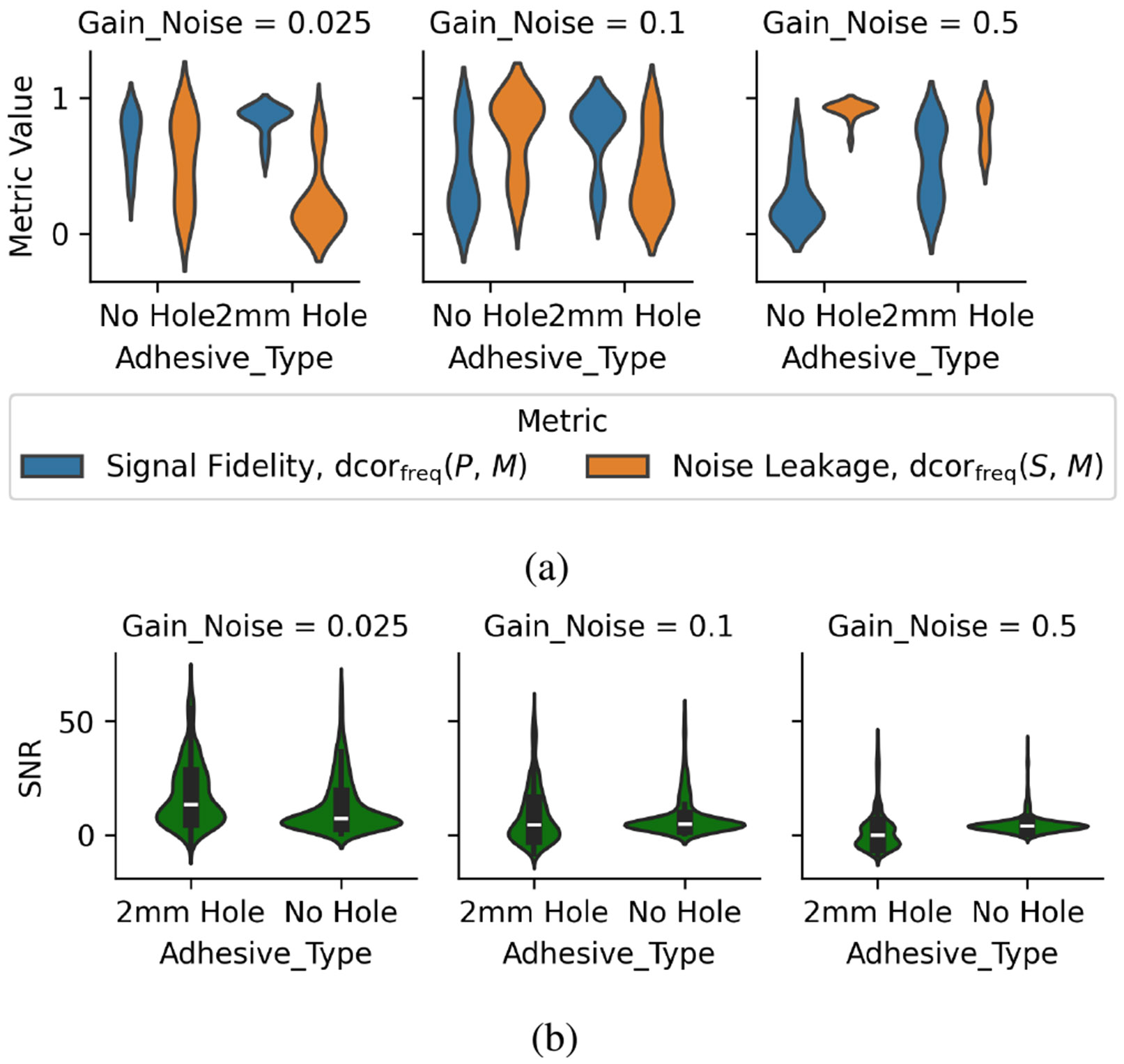
Diaphragms with a hole tend to have better signal fidelity and less noise leakage than solid diaphragms. The visualized conditions evaluate the Cartesian product of four diaphragm materials, four diaphragm thicknesses, and three ambient noise gain levels (corresponding to 57±10, 68±10, and 79±10 dbA). The analyzed frequency range is up to 4 kHz. (a) Signal fidelity and noise leakage and (b) SNR.

The diaphragm attenuates both signal and noise. Summarized in Fig. 5, the evidence suggests that, across all tested diaphragms, a 2 mm hole in the center of the diaphragm tends to result in a stethoscope with higher signal fidelity and lower noise leakage than a solid diaphragm. The result holds for all levels of ambient noise, though when the ambient noise gain is 0.5, corresponding to 79 dbA, a lot of ambient noise leaks into the auscultated signal.

**Fig. 6.**
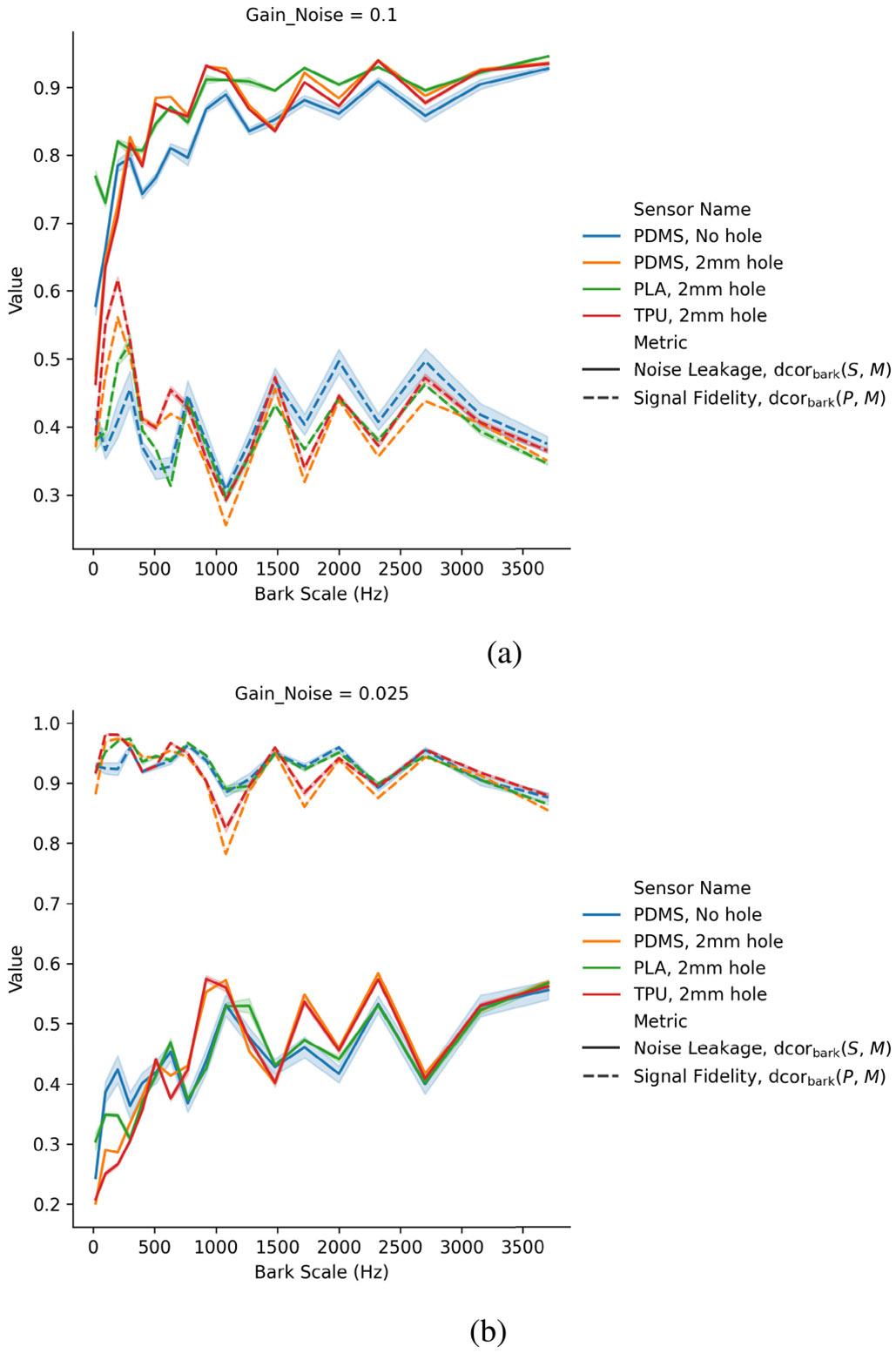
Windowed distance correlation across frequency bands of the Bark scale. The top figure analyzes experiments with ambient noise signals of approximately 68 dbA (gain v=0.1). The bottom figure considered ambient noise at approximately 57 dbA (gain v=0.025). Showing the five sensors with highest sensor quality at each respected ambient noise gain. The variation shows performance differences across diaphragm thicknesses. (a) Ambient noise at 68 dbA (gain 0.1). (b) Ambient noise at 57 dbA (gain 0.025).

Ambient noise distorts the low frequencies more than high frequencies in the tested stethoscopes. Fig. 6 visualizes the amplitude spectra using the critical bands of the Bark scale (less than 4 kHz) on x-axis and distance correlation for each band on the y-axis. It represents how a human would perceive the sound across the frequency spectrum. The ideal representation would have, for any given sensor, a noise leakage of zero, and a signal fidelity of 1 (both horizontal lines). The spectra are a function of the particular electret microphone sensor utilized and the experimental conditions. Each plot shows the sensors with highest quality at the given ambient noise gain. The signal fidelity is consistently higher than noise leakage when the ambient noise has a gain of 0.025 (approximately 57 dbA), and it is lower than the signal fidelity at ambient gain 0.1, or approximately 68 dbA, which is as loud as a vacuum cleaner or the television audio. The curves give a sense for which frequencies the sensors are good listening to. For instance, in the presence of higher ambient noise setting [Fig. 6(a)], the analyzed stethoscopes lose signal fidelity and gain noise leakage at low frequencies, whereas in the low ambient noise setting [Fig. 6(b)], the low frequencies are well captured. It is not known how this phenomenon would generalize outside of this study.

Sensor selection based on human hearing can give different results than sensor selection based on computational analysis though it would be ideal if the two approaches mostly agree Table II shows the highest quality sensors, at different noise levels, as computed by two different quality metrics: $Q_{\text{freq}}$ defined in (3) and a variation of the metric, $Q_{\text{bark}}$, that defines frequency bands based on human hearing (4). The results show that the PDMS diaphragm with 0.5 mm thickness and no hole has the highest signal quality to a human ear, while the TPU diaphragms with a 2 mm hole have best signal quality otherwise. Table I shows that PDMS diaphragms have a high variability of signal fidelity and noise leakage. This variation may be a result of the way the PDMS diaphragms were attached to the enclosure with a TPU retaining ring and perhaps the retaining ring introduced a strain on the PDMS. Due to the variation, it is possible that the 0.5-mm thick PDMS results are an outlier. The TPU with diaphragms 0.5 and 1.5 mm are among the top five sensors for any noise gain and using either quality metric. Additionally, the $Q_{\text{freq}}$ consistently selects TPU material for either noise level, while $Q_{\text{bark}}$ is not consistent in its selection of material. $Q_{\text{bark}}$ also shows lower quality scores than $Q_{\text{freq}}$ in the presence of any noise.

Similarity of signal fidelity and noise leakage statistics with the SNR. Fig. 5 demonstrates that the proposed signal fidelity and noise leakage statistics agree, over the distribution of all experiments, with the SNR. The figure also shows that the SNR is a significantly less explanatory metric. The SNR reduces the power of an observed microphone signal, across analyzed frequencies, to a scalar value, and similarly reduces the power of the corresponding theoretical noise signal to a scalar value. In contrast, the signal fidelity and noise leakage statistics defined over the frequency domain consider the linear and nonlinear relationships between all experiments and all frequencies at once. The two metrics also describe how much the observed signal is related to either the theoretical noise signal or to the theoretical phantom signal. Compared qualitatively to the SNR, at each gain level, both statistics in Fig. 5(a) have less overlap over distribution of across the hole versus no hole than the SNR in Fig. 5(b).

Impedance matching does not capture the full picture. Table III reports the specific characteristic acoustic impedance of the tested diaphragm materials as well as the auscultated surface material on the phantom. The reported density was measured three times with one water bath at 21 °C. Each velocity number in the table is an average of three measurements, each taken using a different water baths. The water bath temperatures were 23.3 °C, 28.3 °C, and 36.7 °C, and no clear trends were observed since the speed of water was adjusted to the water temperature. The samples were submerged in each water bath for several minutes prior to each measurement. We note that the water used in the experiments was contaminated by rust particles from the welded metal platform and screws that supports the ultrasound probes. The presence of rust might increase the speed of sound in water, and the computed acoustic velocity measurements might be slightly low. However, we did not detect an obvious change in velocity when the water bath was one week old (and yellow colored) versus one hour old (and nearly clear). Moreover, our measurements for the PDMS sample (1.16 MRayls) are similar to Rennoll et al. [9] who used the same setup (without rust) and reported PDMS in the range of 1.02–1.58 MRayls.

Table III can be compared to the no hole experiments of Table I. In particular, the no hole diaphragm materials ordered by best-to-worst sensor quality (or signal fidelity or noise leakage) are: PDMS, TPU, Elastic50a, and PLA. If impedance matching is relevant to sensor quality, then the materials with impedance between the phantom’s gelatin layer and the electret microphone’s air column should have the highest quality. The results show that PDMS and Elastic50a have impedances between air and gelatin, but Elastic50a has low sensor quality and signal fidelity, while PDMS has high quality and fidelity, and neither has as good sensor quality as TPU. Perhaps, the difference can be explained by the fact that the PDMS diaphragms were encased in a TPU retaining ring, which provided noise isolation, and the Elastic50a diaphragm wrapped around the base of the enclosure (see Fig. 1), and did not benefit from noise isolation with TPU. The fact that PLA has the largest impedance and lowest sensor quality does suggest that impedance matching is somewhat beneficial for sensor selection, but the results also suggest that impedance matched diaphragm materials can be sensitive to ambient noise. Moreover, impedance matching considers a single interface between two materials, while the diaphragms analyzed consider two interfaces and three materials between the phantom surface, diaphragm material, and air column.

### High-quality digital stethoscope for less than U.S. $5:

The electret stethoscope design in this article offers an inexpensive design to optimize auscultation. The electret microphone costs U.S. $1.25. The TPU enclosure and diaphragm costs approximately U.S. $0.12. The cost of adhesive (3M 2477P), silicone glue, or encapsulant and audio wire is highly variable, but perhaps less than U.S. $3.50. The material cost of a complete stethoscope that connects to a computer via audio cable is therefore approximately less then U.S. $5. Regarding tools, a 3-D printer and soldering station are necessary, and optionally a laser cutter is useful for cutting the adhesive.

## V. Discussion and Future Work

While the acoustic impedance matching literature emphasizes the function of a diaphragm to minimize attenuation of the auscultated signal, our results suggest that another important function of the diaphragm can be to attenuate ambient noise. The results of this article suggest that the absence of material between the skin and electret sensor’s air column can be preferable to the presence of an interface material when considering both noise leakage and signal fidelity. This experimental finding is limited in scope to electret microphones, which are more sensitive to ambient noise than other transducer types.

The acoustic impedance matching literature does not provide a solution for how to design a diaphragm in the presence of two interfacing surfaces, such as would be present in a stethoscope that has a diaphragm and an air column. The literature emphasizes selecting a diaphragm material that approximates the acoustic impedance of the skin. Our results support the selection of a sound attenuating diaphragm material such as TPU, and a small hole in the center of the diaphragm to extend the air column. While impedance matching may have some effect on diaphragm material selection, it may not be as relevant as the material’s ability to suppress ambient noise. Indeed, the PDMS diaphragms demonstrated strong performance (e.g. rank 2 quality score in Table I), but the PDMS was fixed into position by a TPU retaining ring, and the combination of both materials may explain why PDMS has low noise leakage. Future work may consider diaphragm designs that combine materials to maximize noise isolation and signal pickup, as well as consider how to design continuously variable impedance materials that match to skin on one side of the material, and to air on the other side.

Noise leakage through the seams or connection points between material components may be more important to electret stethoscopes than previously thought. For instance, the experimental results with “No Diaphragm” and 2 mm hole in the adhesive, summarized in Table I, shows that bonding the skin directly to the bell-shaped enclosure (middle component of Fig. 1) gives the highest quality signal. Our interpretation of this result is that the TPU enclosure performs sound isolation and acts equivalently to a TPU diaphragm with a hole. Future work can include designs of single part 3-D printed enclosures, or explore acoustically sealing glues that block out contamination by ambient noise sounds.

The results of this work beg the question, why are there no existing commercial stethoscopes with a hole in the diaphragm? Conventional stethoscopes have an acoustic sound hole in the bell, and the bell is primarily useful for low-frequency sounds, but on the main diaphragm, a hole does not exist. The diaphragm itself can serve multiple beneficial purposes, such as to protect the internal components from liquids, and prevent bacterial contamination because it is simple to clean. The diaphragm also acts as a part of the enclosure and it ensures that there can be a seal between the skin and the stethoscope that eliminates sound. Our results suggest that modern electret stethoscopes could have a hole in the center of the diaphragm, and moreover, that it may be beneficial to replace the air column with PDMS, while utilizing a noise isolating material such as TPU for the remainder of the diaphragm.

The use of distance correlation for the analysis of acoustic signals may have a novel application in this work. The properties guaranteed by the metric are appealing for sensor analysis: a zero correlation denotes statistical independence between two signals, whereas a value of one denotes equality of the signals through a linear affine transform of one of them. Future works on the acoustic analysis of stethoscope sensors may consider adopting the distance correlation metric for its desirable properties.

## VI. Conclusion

We have developed an electret microphone stethoscope that costs less than U.S. $5.00, can be fabricated with a 3-D printer and soldering station, and has a high-quality signal. Our results have demonstrated that a primary purpose of a diaphragm in an electret stethoscope is to attenuate noise, and that a diaphragm designed with a hole enabling direct contact with the sensor’s air column and auscultated surface (skin or phantom) can be preferable to a solid diaphragm. We have shown that the 3-D printed TPU material is preferable to using PDMS, PLA, and Form Labs Elastic50a materials in the presence of ambient noise. We have also shown that the diaphragm thickness is not as relevant to sensor quality as the material type and the presence of a hole. The findings of this analysis are limited to the analysis of stethoscopes made with an electret microphone. Finally, we contribute signal fidelity and noise leakage statistics, and we design sensor quality measures to perform sensor selection.

## Figures and Tables

**TABLE I:** Material Selection: TPU Material With 2 mm Hole Is Preferred, and No Diaphragm Is Preferred

Hole	Material	Signal Fidelity		Noise Leakage		Quality		Rank	
		${\mathrm{dcor}}_{\text{freq}}(P,M)$	${\mathrm{dcor}}_{\text{time}}(P,M)$	${\mathrm{dcor}}_{\text{freq}}(s,m)$	${\mathrm{dcor}}_{\text{time}}(s,m)$	$Q_{\text{freq}}$	$Q_{\text{time}}$	$r(Q_{\text{freq}})$	$r(Q_{\text{time}})$
2mm	Elastic50a	0.69±0.26	0.58±0.07	0.54±0.32	0.53±0.08	0.32	0.27	5	5
	PDMS	0.61±0.29	0.56±0.08	0.60±0.34	0.53±0.08	0.24	0.26	6	6
	PLA	0.67±0.27	0.58±0.06	0.50±0.34	0.51±0.08	0.34	0.28	4	3
	TPU	**0.83±0.13**	**0.62±0.04**	**0.33±0.26**	**0.49±0.07**	**0.56**	**0.32**	**1**	**1**
	No Diaphragm	0.74±0.26	0.60±0.07	0.47±0.34	0.52±0.09	0.39	0.29	3	2
No	Elastic50a	0.37±0.28	0.51±0.06	0.81±0.25	0.61±0.06	0.07	0.20	8	7.5
	PDMS	0.77±0.16	0.59±0.07	0.44±0.31 **	0.52±0.08**	0.43	0.28	2	4
	PLA	0.29±0.21	0.49±0.03	0.9±0.06	0.64±0.02	0.03	0.18	9	10
	TPU	0.44±0.29	0.51±0.06	0.79±0.21	0.61±0.05	0.09	0.20	7	7
	No Diaphragm*	0.26±0.14	0.50±0.04	0.93±0.03	0.64±0.03	0.02	0.18	10	9

Note 1: Showing the mean ± standard deviation of the statistic across ambient noise gains and diaphragm thicknesses. A material with low standard deviation is invariant to ambient noise.

Note 2: The best and second best values in each column are boldfaced and underlined, respectively.

* No hole, No Diaphragm - In this experiment only, the double-sided adhesive itself did not have a hole, and it separated the air column from the phantom. In all other experiments, the adhesive always had a hole regardless of whether the diaphragm itself had a hole, as shown in Figure 2.

** The row, No Hole, PDMS shows low noise leakage values, and these may be due to the fact that the PDMS diaphragm was held in place by a TPU retaining ring.

**TABLE II:** Choosing Sensors for Varying Amounts of Noise The Bark scale identifies critical bands of human hearing. $Q_{\text{bark}}$, from Equation (4), captures a sensor’s quality based on the ${\mathrm{dcor}}_{\text{freq}}(\cdot ,r)$ in each of the critical bands in the frequency range from 0 to 4 kHz.

Top Sensor for Any Noise (averaged over all gains v∈𝒱, or 57±10, 68±10, and 79±10 dbA )						
Material	Thickness	Hole	$Q_{\text{bark}}$	$Q_{\text{freq}}$	$r(Q_{\text{bark}})$	$r(Q_{\text{freq}})$
TPU	1.00	2mm	0.202	0.627	5	1
TPU	0.50	2mm	0.205	0.627	4	2
TPU	1.50	2mm	0.214	0.627	2	3
PLA	2.00	2mm	0.179	0.614	12	4
PLA	0.50	2mm	0.163	0.606	14	5
–	0.00	2mm	0.184	0.601	11	6
PLA	0.50	2mm	0.195	0.571	8	7
PDMS	0.50	No	0.241	0.561	1	8
PDMS	2.00	2mm	0.200	0.536	7	9
PDMS	0.90	2mm	0.148	0.533	16	10
PLA	1.00	2mm	0.201	0.528	6	11
PDMS	1.00	No	0.185	0.478	10	12
PDMS	1.50	No	0.209	0.370	3	13
Top Sensors for Low Noise (at gain v=0.025, or 57±10 dbA)						
Material	Thickness	Hole	$Q_{\text{bark}}$	$Q_{\text{freq}}$	$r(Q_{\text{bark}})$	$r(Q_{\text{freq}})$
TPU	1.50	2mm	0.549	0.646	2	1
TPU	0.50	2mm	0.526	0.646	4	2
TPU	1.00	2mm	0.522	0.645	7	3
PLA	2.00	2mm	0.466	0.634	12	4
PLA	0.50	2mm	0.428	0.625	15	5
–	0.00	2mm	0.481	0.624	11	6
PLA	0.50	2mm	0.524	0.600	5	7
PDMS	0.50	No	0.613	0.593	1	8
PDMS	2.00	2mm	0.520	0.576	8	9
PDMS	0.90	2mm	0.391	0.572	16	10
PLA	1.00	2mm	0.537	0.567	3	11
PDMS	1.00	No	0.483	0.530	10	12
TPU	2.00	2mm	0.285	0.445	20	13

**TABLE III:** Acoustic Impedance Matching of Materials

Material	Density, ρ, (kg/m^3^)		Velocity v, (m/s)	Acoustic Impedance z=vρ, ($\frac{\text{kg}}{m^{2}s}$)
	Reference	Measured		
Elastic50a	1010 [37]	1057	1510	1.16e6
PDMS	1030 [38]	1040	1095	1.14e6
PLA	1240 [39]	1200	1934	2.32e6
TPU	1220 [40]	1154	1420	1.64e6
Phantom*	967*	–	1457*	1.41e6
Air, 25°C	1.18	–	346	0.0004e6

* Using gelatin layer (Humimic Medical, Gelatin 2) to simulate skin. The values ρ and v are reported by the manufacturer, and z obtained using the reported values.

## Footnotes

1 Registered trademark.

2 Trademarked.

## References

[1] Sessler GM and West JE, “Foil-electret microphones,” J. Acoust. Soc. Amer, vol. 40, no. 6, pp. 1433–1440, Dec. 1966.

[2] Smith C, “Transducer for sensing body sounds,” U.S. Patent U.S. 6 661 897 B2, Dec. 9, 2003. [Online]. Available: https://patents.google.com/patent/US6661897B2/en

[3] Ji Z and Zhang M, “Highly sensitive and stretchable piezoelectric strain sensor enabled wearable devices for real-time monitoring of respiratory and heartbeat simultaneously,” Nanotechnol. Precis. Eng, vol. 5, no. 1, p. 013002, Mar. 2022.

[4] Chen H , “A two-stage amplified PZT sensor for monitoring lung and heart sounds in discharged pneumonia patients,” Microsystems. Nanoeng, vol. 7, no. 1, p. 55, Jul. 2021. 10.1038/s41378-021-00274-x PMC8433369 34567768

[5] Kumar A , “Recent development and futuristic applications of MEMS based piezoelectric microphones,” Sens. Actuators A, Phys, vol. 347, Nov. 2022, Art. no. 113887. [Online]. Available: https://www.sciencedirect.com/science/article/pii/S0924424722005222

[6] Hu Y and Xu Y, “An ultra-sensitive wearable accelerometer for continuous heart and lung sound monitoring,” in Proc. Annu. Int. Conf. IEEE Eng. Med. Biol. Soc, Aug. 2012, pp. 694–697. 10.1109/EMBC.2012.6346026 23365987

[7] Gupta P, Wen H, Di Francesco L, and Ayazi F, “Detection of pathological mechano-acoustic signatures using precision accelerometer contact microphones in patients with pulmonary disorders,” Sci. Rep, vol. 11, no. 1, p. 13427, Jun. 2021. 34183695 10.1038/s41598-021-92666-2 PMC8238985

[8] Liu Z, Li H, Shi B, Fan Y, Wang ZL, and Li Z, “Wearable and implantable triboelectric nanogenerators,” Adv. Funct. Mater, vol. 29, no. 20, May 2019, Art. no. 1808820.

[9] Rennoll V , “Electrostatic acoustic sensor with an impedance-matched diaphragm characterized for body sound monitoring,” ACS Appl. Bio Mater, vol. 6, no. 8, pp. 3241–3256, Aug. 2023, doi: 10.1021/acsabm.3c00359. PMC10804910 37470762

[10] Schwartz RS, Reeves JT, Sodal IE, and Barnes FS, “Improved phonocardiogram system based on acoustic impedance matching,” Amer. J. Physiology-Heart Circulatory Physiol, vol. 238, no. 4, pp. H604–H609, Apr. 1980. 10.1152/ajpheart.1980.238.4.H604 7377335

[11] Joyashiki T and Wada C, “Validation of a body-conducted sound sensor for respiratory sound monitoring and a comparison with several sensors,” Sensors, vol. 20, no. 3, p. 942, Feb. 2020. 32050716 10.3390/s20030942 PMC7038963

[12] Ertel PY, Lawrence M, Brown RK, and Stern AM, “Stethoscope acoustics,” Circulation, vol. 34, no. 5, pp. 889–898, Nov. 1966. [Online]. Available: https://www.ahajournals.org/doi/abs/10.1161/01.CIR.34.5.899 5923851 10.1161/01.cir.34.5.889

[13] Wodicka GR, Kraman SS, Zenk GM, and Pasterkamp H, “Measurement of respiratory acoustic signals: Effect of microphone air cavity depth,” Chest, vol. 106, no. 4, pp. 1140–1144, 1994. 7924486 10.1378/chest.106.4.1140

[14] Nowak LJ and Nowak KM, “An experimental study on the role and function of the diaphragm in modern acoustic stethoscopes,” Appl. Acoust, vol. 155, pp. 24–31, Dec. 2019. [Online]. Available: https://www.sciencedirect.com/science/article/pii/S0003682X18311265

[15] Nussbaumer M and Agarwal A, “Stethoscope acoustics,” J. Sound Vib, vol. 539, pp. 117–194, Jun. 2022.

[16] Ertel PY, Lawrence M, Brown RK, and Stern AM, “Stethoscope acoustics: II. Transmission and filtration patterns,” Circulation, vol. 34, no. 5, pp. 899–909, Nov. 1966. 5923852 10.1161/01.cir.34.5.899

[17] Nowak KM and Nowak LJ, “Experimental validation of the tuneable diaphragm effect in modern acoustic stethoscopes,” Postgraduate Med. J, vol. 93, no. 1103, pp. 523–527, Sep. 2017. 10.1136/postgradmedj-2017-134810 28289149

[18] Rennoll V, McLane I, Elhilali M, and West JE, “Optimized acoustic phantom design for characterizing body sound sensors,” Sensors, vol. 22, no. 23, p. 9086, Nov. 2022. 36501787 10.3390/s22239086 PMC9735779

[19] Mangion K, “The stethoscope,” Malta Med. J, vol. 19, no. 2, pp. 41–44, 2007. [Online]. Available: https://www.um.edu.mt/library/oar/handle/123456789/803

[20] Leng S, Tan RS, Chai KTC, Wang C, Ghista D, and Zhong L, “The electronic stethoscope,” Biomed. Eng. Online, vol. 14, no. 1, pp. 1–37, 2015. 26159433 10.1186/s12938-015-0056-y PMC4496820

[21] Lee SH, Kim Y-S, and Yeo W-H, “Advances in microsensors and wearable bioelectronics for digital stethoscopes in health monitoring and disease diagnosis,” Adv. Healthcare Mater, vol. 10, no. 22, Nov. 2021, Art. no. 2101400. 10.1002/adhm.202101400 34486237

[22] Zhou S , “Recent advances in TENGs collecting acoustic energy: From low-frequency sound to ultrasound,” Nano Energy, vol. 129, Oct. 2024, Art. no. 109951.

[23] Hui X , “Acoustically enhanced triboelectric stethoscope for ultrasensitive cardiac sounds sensing and disease diagnosis,” Adv. Mater, vol. 36, no. 29, Jul. 2024, Art. no. 2401508. 10.1002/adma.202401508 38747492

[24] Rathod VT, “A review of acoustic impedance matching techniques for piezoelectric sensors and transducers,” Sensors, vol. 20, no. 14, p. 4051, Jul. 2020. 32708159 10.3390/s20144051 PMC7411934

[25] Bakhoum EG and Cheng MH, “Novel electret microphone,” IEEE Sensors J., vol. 11, no. 4, pp. 988–994, Apr. 2011.

[26] Lee SH , “Fully portable continuous real-time auscultation with a soft wearable stethoscope designed for automated disease diagnosis,” Sci. Adv, vol. 8, no. 21, p. 5867, May 2022. [Online]. Available: https://www.science.org/doi/abs/10.1126/sciadv.abo5867 10.1126/sciadv.abo5867 PMC9132462 35613271

[27] Zanartu M, Ho JC, Kraman SS, Pasterkamp H, Huber JE, and Wodicka GR, “Air-borne and tissue-borne sensitivities of bioacoustic sensors used on the skin surface,” IEEE Trans. Biomed. Eng, vol. 56, no. 2, pp. 443–451, Feb. 2009. 19272887 10.1109/TBME.2008.2008165

[28] Semmlow JL, “Improved heart sound detection and signal-to-noise estimation using a low-mass sensor,” IEEE Trans. Biomed. Eng, vol. 63, no. 3, pp. 647–652, Mar. 2016. 26302504 10.1109/TBME.2015.2468180

[29] Székely GJ, Rizzo ML, and Bakirov NK, “Measuring and testing dependence by correlation of distances,” Ann. Statist, vol. 35, no. 6, pp. 2769–2794, Dec. 2007, doi: 10.1214/009053607000000505.

[30] Kundrata J, Fujimoto D, Hayashi Y, and Barić A, “Comparison of Pearson correlation coefficient and distance correlation in correlation power analysis on digital multiplier,” in Proc. 43rd Int. Conv. Inf., Commun. Electron. Technol. (MIPRO), Sep. 2020, pp. 146–151.

[31] Castro-Prado F, Gonzàlez-Manteiga W, Costas J, Facal F, and Edelmann D, “Tests for categorical data beyond Pearson: A distance covariance and energy distance approach,” 2024, arXiv:2403.12711 10.1002/bimj.70129 PMC13244122 42252863

[32] Rocha BM , “An open access database for the evaluation of respiratory sound classification algorithms,” Physiol. Meas, vol. 40, no. 3, Mar. 2019, Art. no. 035001. 30708353 10.1088/1361-6579/ab03ea

[33] McCollum ED , “Digital auscultation in PERCH: Associations with chest radiography and pneumonia mortality in children,” Pediatric Pulmonol., vol. 55, no. 11, pp. 3197–3208, Nov. 2020. 10.1002/ppul.25046 PMC7692889 32852888

[34] Zwicker E, “Subdivision of the audible frequency range into critical bands (Frequenzgruppen),” J. Acoust. Soc. Amer, vol. 33, no. 2, p. 248, Feb. 1961.

[35] Lubbers J and Graaff R, “A simple and accurate formula for the sound velocity in water,” Ultrasound Med. Biol, vol. 24, no. 7, pp. 1065–1068, Sep. 1998. 9809641 10.1016/s0301-5629(98)00091-x

[36] Hughes SW, “Archimedes revisited: A faster, better, cheaper method of accurately measuring the volume of small objects,” Phys. Educ, vol. 40, no. 5, pp. 468–474, Sep. 2005.

[37] Safety Data Sheet for Biomed Elastic 50a V1, Form Labs, Somerville, MA, USA, Sep. 2023.

[38] Luo X and Mather PT, “Preparation and characterization of shape memory elastomeric composites,” Macromolecules, vol. 42, no. 19, pp. 7251–7253, Oct. 2009.

[39] Technical Data Sheet for Pla Basic Filament, Bambu Lab, Shenzhen, China, Sep. 2024.

[40] Technical Data Sheet for Tpu 95a Hf Filament, Bambu Lab, Shenzhen, China, Sep. 2024.

